# Synthetic lethality by PARP inhibitors: new mechanism uncovered based on unresolved transcription-replication conflicts

**DOI:** 10.1038/s41392-024-01893-2

**Published:** 2024-07-29

**Authors:** Marina Kolesnichenko, Claus Scheidereit

**Affiliations:** 1 Department of Hepatology and Gastroenterology, Charité-Universitätsmedizin Berlin, Berlin, Germany; 2 Laboratory for Signal Transduction in Tumor Cells, Max Delbrück Center for Molecular Medicine in the Helmholtz Association (MDC), Berlin, Germany

**Keywords:** Drug development, Cancer genetics

**Fig. 1:**
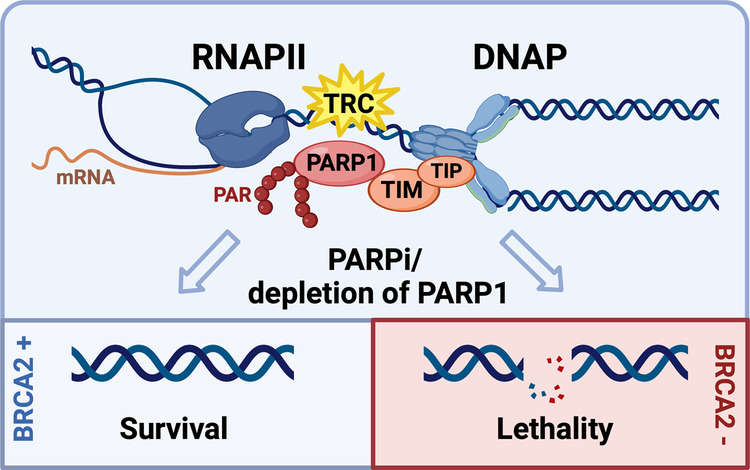
New mechanism for synthetic lethality mediated by PARP inhibitors. TRCs are a major cause of genome instability and DNA double-strand breaks (DSBs). Conflicts occur when DNA replication forks and elongating RNA polymerases collide and require PARP1, TIMELESS (TIM) and TIPIN (TIP) for resolution. PARP and TIM interact and signal TCR to the replisome. After binding to DNA lesions, PARP1 is activated and forms poly(ADP)-ribose (PAR) attached to itself and to other proteins. PARP1 inhibition or loss of PARP1, TIM or TIP results in the generation of DSBs which, in the absence of homologous repair capacity, result in cell death

In a landmark study recently published in *Nature*, Petropoulous et al. demonstrated that synthetic lethality of poly(ADP-ribose) polymerase (PARP) inhibitors in cells with defective homologous recombination repair (HR) results predominantly from transcription replication conflicts (TRC) and not, as previously proposed, from PARP trapping on DNA^1^ (Fig. 1). The article unveils a new mechanism behind synthetic lethality of PARP inhibitors with relevance for cancer therapy.

DNA damage response (DDR) networks maintain genome integrity in the cell. Cancer cells frequently accumulate mutations in DDR genes and targeting DDR can induce excessive DNA damage. Synthetic lethality refers to the combination of a mutated gene, e.g. responsible for DNA repair, with the inactivation of another gene or its product, which together are toxic and induce cell death. PARP1/2 inhibitors emerged in the past decade as prominent blockers of HR-mediated repair of double-strand DNA breaks (DSB) and four are now available worldwide for treatment of breast, prostate, and pancreatic cancers.^2,3^

Of the 17 PARP family members, only PARP1 and 2 function in HR. As the most active enzyme, PARP1 binds to damaged DNA and facilitates the recruitment of chromatin remodelling and DNA repair effectors through poly-ADP-ribosylation (PARylation) of substrates. Eventually, PARP1 auto-PARylation causes the release of PARP1 from DNA. PARP1/2 inhibitors were believed to function by trapping these proteins on DNA, thereby leading to accumulation of DNA damage due to replisomes colliding with the DNA-bound PARPs.^3^ PARP1/2 trapping has been linked with cytotoxicity, particularly to occurrence of anaemia and leukaemia. Trapping may also prevent PARPs from mediating numerous other functions, including chromatin remodelling, transcription, and regulation of numerous pro-inflammatory and metabolic processes. Significant cellular toxicity from broad inhibition of PARPs could arise from interference with its other physiological functions in normal cells. This makes it necessary to identify more selective PARP inhibitors that only block those functions that permit cancer cells to survive.

PARP1 interacts with TIMELESS and TIPIN, which were previously shown in yeast to protect the replisome from conflicts with transcription. The authors demonstrated that in human cells TIMELESS and TIPIN likewise prevent TRC. Their depletion leads to DNA damage, which critically, depends on active DNA replication in the early S-phase of the cell cycle and on ongoing transcription. Direct interaction between PARP1 and TIMELESS averts TRC and subsequent DNA damage, presumably via PARP1 signalling the presence of TRC to the replisome. Depletion of PARP1 phenocopies depletion of TIMELESS and TIPIN, confirming that the proteins function in the same pathway.^1^ This suggests inhibiting any of the three proteins suffices for synthetic lethality in the context of HR deficiency.

The DNA trapping capacities of the different PARP inhibitors inversely correlate with the maximum tolerated dose in the clinic.^2^ The authors found that the efficacy of PARP inhibitors in inducing TRC-dependent DDR in the S-phase of HR-deficient cells did not correlate with their trapping capacity but rather with the inhibition of PARP1 enzymatic activity.^1^ Likewise, the depletion of PARP1, but not PARP2, triggered DDR, which was dependent on ongoing transcription in the S‐phase. Since depleted PARPs cannot be trapped, the authors concluded that inhibiting PARP1 enzymatic activity is sufficient to impair TRC resolution.^1^ As previously shown, toxicity of PARP inhibitors correlates with their trapping capacity.^2^ The findings of Petropoulous et al. therefore open novel means for decreasing toxicity without sacrificing efficacy by reducing the trapping potential of PARP inhibitors.

To demonstrate that TRCs in the context of HR deficiency drive synthetic lethality, the authors tested PARP inhibitors in HR-deficient, Breast Cancer Susceptibility Gene 2 (*BRCA2-/-*) cells. BRCA1 and 2, frequently mutated in breast and ovarian cancers, are key players in HR-mediated DNA repair. Unlike normal cells, *BRCA2-/-* cells accumulated DNA damage in S-phase, dependent on active transcription. TRC-dependent synthetic lethality was achieved only in *BRCA2-/-* cells, indicating that unresolved TRCs due to defects in HR account for the selective killing of *BRCA2-/-* cells treated with PARP inhibitors.^1^ Importantly, depletion of PARP1 compromised survival of *BRCA2-/-* cells in a manner similar to PARP inhibitors (Fig. 1), confirming that impaired PARP1 enzymatic activity, and not trapping, drives synthetic lethality associated with HR deficiency.

The key objective of therapeutic synthetic lethality is to eliminate cancer cells, while leaving normal cells intact. To test how killing of *BRCA2-/-* versus normal *BRCA2+/+* cells is affected by DNA trapping capabilities of the different PARP inhibitors, the authors performed dose–response analyses. Strikingly, increased DNA trapping of PARP1 decreased selectivity for HR-deficient cells and led to an almost 200-fold increase in the killing of normal cells.^1^ These findings underscore the importance of targeting enzymatic activity of PARP1 and reducing trapping potential of future inhibitors.

In summary, the authors propose a new model for synthetic lethality of PARP inhibitors in the context of HR deficiency: TRCs are detected by PARP1, which signals to TIMELESS, thereby stalling the replisome until TRCs are resolved. This prevents collisions between replication and transcription machineries and averts accumulation of DNA damage and cell death. In the absence of PARP1 or upon inhibition of its catalytic activity, TRCs remain unresolved and elicit DNA damage that cannot be repaired in HR-deficient cells, resulting in cell death. These insights may lead to the development of novel therapeutic agents selectively inhibiting PARP1 without causing PARP trapping. Furthermore, this work heralds exploration beyond PARPs for synthetic lethality in combination with HR deficiency. Targeting TIMELESS or other components that enable sensing of TRC could dramatically expand the repertoire and the reach of possible targets.

Nonetheless, the mechanism behind sensing and transducing the presence of TRC remains largely unexplored. How do PARP1 and TIMELESS communicate? Another recent study^4^ found that PARP1 PARylates TIMELESS, whose proteasomal degradation then facilitates fork stability.^4^ How does PARP balance its role in DNA repair versus in transcription? PARP1 regulates transcription of anti‐apoptotic genes in a PARP1- and NF‐κB‐dependent manner; however, little is known about the effect of PARP inhibitors on transcription-related functions of PARP1 and their contribution to the cytotoxicity of PARP inhibitors.^5^

In summary, the data of Petropoulos et al. propose novel strategies for drug discovery for malignancies associated with HR-deficiencies, which now await validation in pre-clinical and clinical studies across different cancer types.
